# Patient and Provider Experiences With Precision Oncology: Qualitative Descriptive Study at the Department of Veterans Affairs

**DOI:** 10.2196/84858

**Published:** 2026-04-27

**Authors:** Daniel Becker, Kenneth Csehak, Alexander Barbaro, Christian Miller, Antoinette Vo, Stefanie Roman, Danil Makarov, Scott Sherman, Allison Squires

**Affiliations:** 1 VA NYHHS (Veterans Affairs New York Harbor Healthcare System), New York, NY, United States; 2 NYU Langone, 550 First Avenue, New York, NY, 10016, United States, 1 212-263-2868; 3 Northwell Health, New York, NY, United States

**Keywords:** oncology, precision oncology, health services, qualitative, health equity

## Abstract

**Background:**

Precision oncology (PO) improves and extends the lives of patients living with cancer, but multiple studies have documented its underuse in practice. Specifically, studies note a significant lack of PO use within the Veterans Affairs (VA) medical system. A paucity of implementation of PO in oncologic practice poses a significant barrier to providing the most up-to-date guideline-based care.

**Objective:**

While several studies have explored determinants of PO use, we sought to contribute to the body of knowledge by additionally focusing on the unique perspectives of patients, as well as conducting a comprehensive study within the VA medical system, the United States’ largest single-payer health care system. We conducted interviews with both patients and providers at multiple VA sites to identify and characterize barriers and facilitators of PO use in clinical care.

**Methods:**

Using a qualitative descriptive approach, we conducted semistructured interviews with 17 patients with cancer and 16 oncology providers recruited from multiple VA sites. Cancer types included prostate, gastrointestinal, and lung. Data were analyzed via a team-based coding approach using directed content analysis. Data were coded and then aggregated into themes and mapped to the Theoretical Domains Framework (TDF) and Behavior Change Wheel sources of behavior (Capability, Opportunity, and Motivation) based on the consensus of the study team.

**Results:**

The patient sample consisted of 17 all-male veterans seen at VA oncology clinics in 2022. Participants predominantly self-identified as White (n=9, 52.9%) or Black (n=6, 35.3%), and the majority (n=11, 64.7%) held a high school degree or a higher level of education. The provider sample consisted of 16 physicians, all of whom held MD degrees and practiced oncology. The provider sample represented 6 states, was 50% (8/16) female, and participants averaged 14 years in their current position. The overarching theme was the “Precision Oncology Feedback Loop,” which captured the essence of the complex processes involved in facilitating PO care in the VA system. The TDF and Behavior Change Wheel helped categorize findings to identify where issues in the feedback loop could facilitate or generate barriers to care.

**Conclusions:**

Our findings expand on the current literature by highlighting both patient and provider experiences across key TDF domains (Environmental Context and Resources, Knowledge, Memory, and Attention). The conceptual model produced by the analysis illustrates the complexities associated with the implementation. Our findings support the design of multilevel interventions that target increased knowledge or education, improved workflow, and ease of communication to enhance PO delivery.

## Introduction

Precision oncology (PO), the identification of tumor-specific abnormalities as targets for personalized therapy, has revolutionized cancer care over the last several decades by extending survival and minimizing treatment-related toxicities [1]. Despite the benefits established in clinical trials, extensive studies by our group and others document that up to 50% of patients do not receive guideline-recommended PO treatment. For example, molecular testing of colon cancer biopsy tissue for clinically targetable alterations in patients with advanced colon cancer has been recommended by expert consensus guidelines since 2010 [2]. Studies of this population in the Veterans Affairs (VA) health system established that such testing was completed in only 28% of eligible patients with advanced colon cancer by the year 2015 [3]. This hinders the ability of clinicians to use the cancer mutational profile to determine therapy, which is standard care for patients with advanced colon cancer. Similarly, among patients with non-VA colorectal cancer included in the Surveillance Epidemiology and End Results database, molecular testing was completed for only 31% of eligible patients [4,5].

Consistent with ecological models of health behaviors, barriers and facilitators to PO exist at many levels (eg, patient, provider, and system) and steps in the process of care [6,7]. At the patient level, older patients and those with Medicaid insurance are less likely to receive PO [3,8]. Furthermore, Black and Latino patients have been consistently underrepresented in the practice and study of PO [9]. Furthermore, at the provider level, quantitative and chart review data suggest that providers do not always act upon PO testing results [10]. Facility and systems-level barriers have also been identified in multiple studies, including lower testing rates in lower-volume cancer centers, difficulty coordinating testing, financial barriers, and lack of clear protocols for test indications and use [10,11].

A recent review of qualitative research on barriers and facilitators to PO suggests that barriers often involve insurance or reimbursement as well as a lack of certainty with regard to clinical utility, while facilitators include the incorporation of PO into clinical guidelines. Notably, this review included only 5 qualitative studies, with very limited representation of oncology clinicians and patients with cancer [12]. One study included no clinicians, another included only 1 clinician, and a third included only 3 clinicians [13-15]. The final 2 studies included 10 and 14 clinicians, respectively, but did not include clinicians in the VA [16,17]. Only one study specified the inclusion of patients and included 16 [16]. While this suggests that there is active investigation in the space, there are relatively few studies, with small sample sizes, and no prior VA representation.

The Veterans Health Administration is the largest integrated health system in the United States, and the VA National Oncology Program cares for approximately 43,000 veterans newly diagnosed with cancer annually [18]. We designed a qualitative study of patients with cancer and providers based on the Theoretical Domains Framework (TDF) [19] to explore barriers and facilitators to PO care in this population. We know that molecular testing, essential for the standard of care in cancer treatment, is being underused. The reasons for the underuse of molecular testing remain unclear, and we are conducting a qualitative investigation to facilitate an in-depth exploration of the patient and provider experiences of PO and identify areas in which interventions to increase guideline-concordant PO can be effectively used.

## Methods

### Design

We used a qualitative descriptive approach with directed content analysis to structure the study. The goal of qualitative descriptive studies is to describe the experiences of those participating in the health-related behavior or process under consideration [20]. When used for health services research, this approach can identify and differentiate common and unique factors experienced by patients and clinicians when engaging with health services.

### Theoretical Underpinning

The TDF is a widely used behavior change framework, consisting of 14 domains that are used in the identification of determinants of change [21]. TDF categories are then mapped to the Behavior Change Wheel (BCW) components of Capability, Opportunity, and Motivation. The BCW is an evidence-based framework for understanding drivers of behavior and translating that understanding into actionable change [22]. Both TDF and BCW were chosen for this analysis over other frameworks, such as the Consolidated Framework for Implementation Research. The TDF can be more useful for the design of qualitative research interview guides by specifying discrete domains, which are applicable at the individual level as well as at the systems level. Our focus was predominantly on the behaviors and experiences of the patients and providers, which is best suited to TDF and BCW over the more process-oriented Consolidated Framework for Implementation Research.

### Reflexivity

The research team comprised 4 researchers: one senior qualitative PhD researcher who is a nurse (AS) and three MD researchers board-certified in internal medicine (KC, DB, and AB). Of the MD researchers, one was a practicing oncologist, and two were oncology fellows in the third year of their training. The nurse researcher had no oncology background and brought methodological expertise. All identify as having White European heritage.

### Sample and Setting

Adult patients seen in oncology clinics between January and June 2022 at participating VA medical centers (VAMCs) in Pittsburgh and New York with a histologically confirmed prostate, gastrointestinal, or lung cancer were included in the study. We chose these 3 cancers as they together represent more than 60% of all patients with cancer within the VA, and PO is well established in all 3 cancers. We chose common cancers to understand practice patterns in patients with relatively common oncologic diseases. Patients with other cancers were excluded. The US Department of Veterans Affairs includes both VAMCs with inpatient and outpatient care as well as VA Community–Based Outpatient Care Centers with outpatient care only. Our study was conducted only at VAMCs. The VA is a single-payer health care system that serves 9 million American veterans and veteran family members.

### Recruitment

Prospective patient participants were recruited by either letter or flyer. A letter was mailed to patients at the Veterans Affairs–New York Harbor Health Care System (VA-NYHHS), whereas a flyer was used at the Pittsburgh VAMC. At the Pittsburgh VAMC, flyers were placed in highly visible locations in the oncology clinics, such as the front desk at registration and in patient examination rooms. Providers were encouraged to inform patients that the study was available. The recruitment of provider participants was email-based. Emails were sent to oncology physicians at the VA-NYHHS, Greater Los Angeles Area (GLA), Pittsburgh, Michigan, and Seattle VAMCs. Informed consent was obtained from all participants, with all study methods approved by the local VA institutional review board (IRB). Patients were eligible for this study if they were receiving medical care at one of the participating VAMCs and were receiving treatment for prostate, lung, or gastrointestinal cancer. Only English-speaking patients were eligible, as US military enlistment requires English proficiency. Providers were eligible if they provided care for patients with cancer in one of the participating VAMCs.

### Data Collection

The semistructured interview guide was constructed through an expert review process by a multidisciplinary team of oncologists, qualitative health researchers, and patient representatives. All 14 TDF domains were represented by at least 1 question in the interview tool. Three patient and three provider interviews, not included in the sample population, were used to refine the interview guide and construct a draft code list. This was done to provide test samples for assessing the feasibility of the interview guide.

Two interviewers then conducted semistructured interviews with patients and providers that lasted between 30 and 60 minutes. All interviews were conducted via audio using patients’ telephone numbers through Microsoft Teams. The interviews were transcribed using Microsoft Teams’ automated transcription feature, with verification by study personnel. Interviews continued until data saturation was achieved.

### Data Analysis

Directed content analysis using the TDF categories guided the analytical process. Directed content analysis allows for codes dictated by a preanalysis identified list, as well as purely descriptive codes, to emerge during the process [23].

AS provided coding training to the physician team and reviewed findings as they emerged. A team-based coding approach structured the consensus-based process [24]. Based on guidance from Cascio et al [24], we drew from principles of team-based coding to generate intercoder consensus throughout the analytic process. The consensus process was documented throughout the study to record the process of data review, idea generation, and analytical technique. The consensus process was conducted in person through meetings with the entire coding group.

Atlas TI.web (version 9.24.3; Lumivero) was used to code interviews. Each interview, whether from a patient or clinician, was coded independently by 3 research staff members (DB, KC, and AB). Initial codes came from the theoretical framework to start the process, and others emerged iteratively during the coding process. All team members met after 3 interviews were coded to harmonize code names and conceptual definitions. The codes were then compared and reviewed to achieve consensus on any coded data about which study staff disagreed. Disagreements regarding code application were discussed among the study team until a consensus was reached. A common codebook was then generated, and the team members checked in periodically to continue coding harmonization throughout the process.

The team then met to finalize categories and themes that emerged independently from the data as well as to evaluate how coding aligned with the TDF theoretical framework. Similar code groups were aggregated and mapped to related BCW sources of behavior (Capability, Opportunity, and Motivation) based on the consensus of the study team.

### Ethical Considerations

A determination of minimal risk and approval was granted by the VA-NYHHS IRB for our protocol (1605219‐1). We received subsequent approval from the Research and Development Committee and the Research Safety and Security Subcommittee. Consent forms were reviewed and approved by each committee and subcommittee. Informed consent was obtained from each participant in accordance with IRB-approved documents. Privacy and confidentiality were protected in accordance with data security specifications outlined in the IRB-approved protocol and were further reviewed by the institutional data security officer. Participant compensation consisted of US $25 gift cards for each participant.

## Results

### Overview

At the VA-NYHHS, letters were sent to 136 patients, of whom 10 (7.4%) enrolled. At the Pittsburgh VAMC, flyers were posted for patients attending the oncology clinic. Of these patients, 7 (1.8%) enrolled, from approximately 400 patients who passed through the clinic. A total of 17 patients and 16 providers (n=33) were interviewed.

Patient participants were predominantly aged 70 to 80 years, entirely male, and mostly White. Twenty-four providers received the email, of whom 16 (67%) participated. Of the 16 providers who were interviewed, 1 provider requested to reply to demographic questions by email but did not subsequently respond. Providers were mostly New York–based, predominantly aged 40 to 50 or 50 to 70 years, and predominantly White. Providers were mainly MDs. Patient and provider characteristics are further outlined in Tables1  2 and include self-reported age, gender, race or ethnicity, as well as the state of the site from which the participant was recruited. For providers, additional information was collected regarding the number of years in position, type of professional degree, and leadership role status.

**Table 1.** Provider characteristics collected for the study of barriers and facilitators to precision oncology.

Characteristic	Participants
Number of providers	
Completed interviews	16
State site, n (%)	
California	1 (6.3)
Michigan	2 (12.5)
North Carolina	1 (6.3)
New York	8 (50)
Pennsylvania	2 (12.5)
Washington	1 (6.2)
Not specified	1 (6.2)
Age range (y)	
30‐40	1 (6.3)
40‐50	5 (31.2)
50‐60	3 (18.8)
60‐70	6 (37.5)
Unknown	1 (6.2)
Gender, n (%)	
Male	7 (43.7)
Female	8 (50)
Other	1 (6.3)
Race, n (%)	
White	10 (62.5)
Asian or East Indian	4 (25)
Other	1 (6.3)
Unknown	1 (6.2)
Ethnicity, n (%)	
Hispanic	0 (0)
Not Hispanic	14 (87.5)
Other	1 (6.3)
Unknown	1 (6.2)
Professional degrees, n (%)	
MD	11 (68.7)
MD and MDA	1 (6.3)
MD and MBBS	2 (12.5)
MD and PhD	1 (6.3)
Unknown	1 (6.2)
Years in current position, mean (range)	13.6 (1‐29)
Leadership role, n (%)	
Yes	10 (62.5)
No	5 (31.2)
Unknown	1 (6.3)

**Table 2.** Patient characteristics collected for the study of barriers and facilitators to precision oncology.

Characteristic	Participants, n (%)
Patients	
Completed interviews	17 (100)
State site	
New York	10 (58.8)
Pennsylvania	7 (41.2)
Age range (y)	
50‐60	1 (5.9)
60‐70	2 (11.8)
70‐80	12 (70.6)
80‐90	2 (11.7)
Gender	
Male	17 (100)
Female	0 (0)
Other	0 (0)
Race	
Black	6 (35.3)
White	9 (52.9)
Native American	1 (5.9)
Other	1 (5.9)
Ethnicity	
Hispanic	1 (5.9)
Non-Hispanic	14 (82.4)
Unknown or none	2 (11.7)
Highest education	
Some high school	2 (11.8)
High school graduate	4 (23.5)
GED^a^	1 (5.9)
Some college	2 (11.8)
College graduate	5 (29.4)
Unknown	3 (17.6)

a GED: graduate equivalency degree.

### Overarching Theme: The Precision Oncology “Feedback Loop”

Our findings suggest that there is a cyclical feedback loop related to the delivery of PO. The feedback loop is driven by the opportunity for this kind of care, as dictated by the resources of the system where care is delivered. Satisfaction from the clinician and patient side is the result of technical process outcomes associated with the PO testing process. Motivation to engage with PO services is a product of clinician learning and success with the tool and the resulting success with patient treatments, which also generates happiness for the patient.

**Figure 1.**
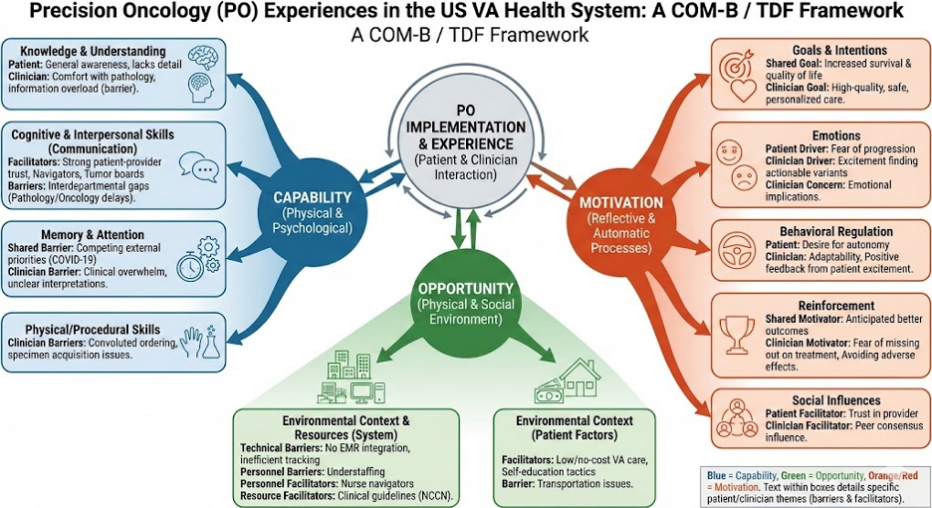
Precision oncology feedback loop. EMR: electronic medical record; NCCN: National Comprehensive Cancer Network; TDF: Theoretical Domains Framework; VA: Veterans Affairs.

The following sections illustrate how the feedback loop occurs, with the TDF providing structure for the categories that shape it. Figure 1 illustrates the feedback loop of Capability, Opportunity, and Motivation on the part of both patients and providers, all of which contribute to patient and provider engagement leading to PO. Sections are organized by the framework’s components.

### Behavioral Regulation (TDF)–Motivation (BCW)

In the TDF, behavioral regulation is defined as the components aimed at managing or changing objectively observed or measured actions, while motivation aligned with the BCW is defined as brain processes that energize and direct behavior [21,22].

In our analysis, statements that referred to self-moderation of actions on the part of patients and providers fell into this category. Patients reflected on the importance of a sense of motivation and autonomy in navigating their care, while providers identified motivation and adaptability as components of effective action. Furthermore, positive feedback emerged as providers sensed that patients were motivated to pursue testing. As one clinician described:

*I think most of the guys get it pretty good and are excited to hear that there’s something beyond standard chemotherapy that they’ve heard all the horror stories about. They’re excited about it so as you move from standard chemotherapy into immunotherapy, now into gene therapy, people are happy to hear that there’s more knowledge, more options, more thinking, we’re getting somewhere with cancer treatment.*

[Provider 13]

### Cognitive and Interpersonal Skills Capability: The Impact of Communication

This category captures the way in which respondents felt communication and coordination between various individuals affected PO and cancer care. Three key motifs emerged, centered on the intersections of multilevel aspects of communication.

#### Interdepartmental Communication

Participants noted the issues that arise, even with an electronic health record, when trying to coordinate departments involved in diagnosis, treatment, and management of care. For example, several respondents identified issues with delayed next-generation sequencing (NGS) results due to gaps in communication between different medical specialties (eg, proceduralists, oncologists, and pathologists):

*It would be nice if pathology had a way to reflexively send the specimen. I guess it’s hard for them. They don’t know the whole situation and you kind of need somebody to oversee it, but that again is just another frustrating thing.*

[Provider 2]

Conversely, several respondents cited regular interdepartmental meetings, such as tumor boards, as helpful in reducing delays.

#### Interdisciplinary Communication

Interactions between providers and nurses, between providers and trainees, and between providers and administrators were all identified as key elements of PO care. Multiple providers also praised an individual coordinator responsible for PO implementation.

#### Patient-Provider Communication

Overall, respondents on both the patient and provider sides expressed that communication with their counterparts was an effective and important facilitator of PO. “The doctors I went to, I think, were extremely thorough in describing what was going and what the treatments were, and answering my questions” (patient 51). On the other hand, a lack of patient understanding was reported as a barrier on the part of clinicians.

### Emotions and Motivation

Emotions and motivation statements reflected the interactions between how patients and clinicians highlighted the role of emotions in influencing PO practice. Unsurprisingly, most of the negative patient-reported emotions reflected fear of their cancer, its progression, and death. Providers cited concerns about the emotional and practical implications of detecting germline mutations.

Nonetheless, finding an actionable mutation was in itself a significant provider motivator:

*I think that there’s kind of a sense of happiness when we find one of those really high yield variants, when you find the EGFR or the ALK in lung cancer. You feel really happy sharing that information with patients and obviously I think they understand that when the provider is happy with something that that’s probably good news for their cancer.*

[Provider 5]

Thus, the balance of emotions and motivations encapsulates the inherent rewards and challenges of the PO treatment process for both clinicians and patients.

### Environmental Context and Resources Shaping Opportunity

#### Overview

Both providers and patients talked extensively about how the care environment and its resources were critical for treatment opportunities. The overall environment and the provider’s ability to navigate the resources to get patients their treatment were essential for balancing patient-level factors that influenced care choices. How they described these aspects of the environment fell into two subcategories of “system-centered” and “patient-centered” descriptions.

#### System: Technical

The organizational structure of the PO process functions was extensively discussed by participants. This included challenges with ordering forms, a lack of dedicated communication channels between pathology and the ordering provider, and inconsistencies in how results are eventually uploaded and displayed. For example, one respondent noted:

*We’ve had to overcome some issues because there is no ordering method within the electronic medical record […] So right now we’re doing this by emails. So essentially, we identify patients in the clinic that need sequencing, and I have a coordinator that is in clinic with me each time I'm in clinic and she will send specimens for sequencing. It’s kind of inefficient.*

[Provider 7]

Among facilitators, patient consent and electronic ordering processes were cited as technical aspects of the system that functioned well and did not delay care delivery.

#### System: Personnel

Respondents cited understaffed provider departments as barriers and navigators and administrative assistants as facilitators. Noting the effectiveness of a nurse navigator working in the system, a provider offered an example of facilitated communication flows:

*The nurse navigator does usually read the report and they will scan and send me an alert, an email, and say “this is someone who’s got an actionable mutation” just to make sure that I've read the report.*

[Provider 12]

Understaffing reflected vacancies, hiring processes, and other factors associated with operating within a government bureaucracy.

#### System: Practice Resources

Clinical and institutional guidelines were noted to help providers recognize PO testing indications. These were seen as important for quickly discerning treatment plans and managing patient responses to them. As one provider stated:

*So my overall goals do align with guidelines, such as NCCN guidelines. So they just hopefully align with standard of care.*

[Provider 4]

For some providers, these guidelines served as educational tools to update their practice.

#### Patient Factors

Logistics, financial challenges, and how patients were educated about their treatments and options were the patient-centered factors that influenced the progression of PO care. Transportation issues were the most frequently cited barrier to care. Many veteran participants were economically vulnerable and did not always have access to reliable public transportation as an alternative. For example, one patient participant stated “sometimes I’d have an appointment that was too early and I’d request transportation and it was hard” (patient 058).

Despite economic vulnerability, the low or no-cost care provided to VA patients was viewed as a significant facilitator that helped ensure veterans were protected from the financial toxicity that can be associated with cancer care outside the VA system. Finally, patients reported a variety of strategies to educate themselves about their diseases and treatments. While some patients mentioned receiving educational materials from their doctor or other VA sources, many more reported using external resources, such as the internet or family members, to learn about their disease and treatment options. One patient stated:

*My wife she goes online … that’s where she learned about the endocarditis because she had to deal with it too. And she’s had sepsis and cancer and she goes to all of the medical sites.*

[Patient 056]

Educating themselves was a clear goal and motivator for patients, which highlights the value of patient engagement in the PO process. They wanted to understand the causes behind their cancer, including potential germline mutations, and to be informed about their testing results and treatment plans.

### Goals: Motivation

Providers had clear goals for PO care that motivated them when working with patients throughout their cancer treatment. They formed into 2 categories focused on patient outcomes and quality of care. Both providers and patients shared a mutual outcome goal of an increase in the length of life or a cure to their disease, as well as their desire to maintain a good quality of life. As one provider stated: “The goals of patient care would be to help people be on the best therapy with the lowest side effects that keeps them alive the longest” (Provider 010). Many provider respondents expressed the importance of the highest quality care, including safety, personalized care, and the ability to treat actionable mutations. They viewed this as inherently intertwined with a good patient outcome.

### Knowledge: Capability

#### Overview

Patients and providers reflected on the role of knowledge in the implementation of PO. Among patients, knowledge of their disease and how it is treated was often broad, with a lack of information constituting a barrier. Conversely, among providers, a deep understanding of both the disease and its treatment facilitated greater engagement in PO.

#### Patient Disease

Although many patients expressed an awareness of genes and their contribution to cancer, more statements were identified in which patients specifically reported a lack of knowledge or understanding about the cause of their disease and its treatment as stated by a patient:

*How it forms is I’m not sure. It’s something to do with your cells*.

[Patient 57]

#### Patient Treatment

Patients frequently expressed knowledge of their treatment in broad, overarching terms, with occasional confusion between modalities of treatment. A lack of familiarity with terms such as “precision medicine” and “targeted treatment” was prevalent. One patient stated:

*I know that everyone has DNA and it’s specific to each person and genes ... I’ve heard of gene therapy but I’m not sure if it apples to cancer*.

[Patient 055]

#### Provider Understanding of Disease and Treatment

Providers expressed comfort with knowledge of the role of mutations in cancer pathogenesis and treatment. They also, however, pointed out the quickly changing PO landscape as a barrier and guidelines and peer-reviewed sources as facilitators. NGS reports themselves were felt to be “a little bit better in terms of outlining what’s pathogenic and what’s not” (provider 11).

### Memory and Attention: Capability

Among both patient and provider participants, external distractors emerged as barriers to PO, with responses generally categorized as prioritization either within the clinic or outside the clinic.

#### Prioritization Within the Oncology Clinic

Some providers expressed difficulty with practicing PO in part because of competing oncologic concerns, such as complex treatment and comorbidity management. Furthermore, providers noted feeling overwhelmed by variable interpretations of PO testing, unclear clinical implications of certain mutations, and excessive extraneous information. Providers noted facilitators, including tumor boards and reflex testing.

#### Prioritization Outside the Oncology Clinic

For patients and providers, the COVID-19 pandemic and associated personal and professional distractions were noted:

*I mean the hardest part is just always just doing too many things at once. Like sometimes Path will just call me and I’ll be there in the middle of clinic. One time I was at softball practice for my 7-year-old and like they're like, “Oh yeah, we didn’t get enough tissue” and my head is in a totally different place, I can’t write that down.*

[Provider 9]

Patients also noted the need to manage other conditions and social issues.

### Skills: Capability

For the second least-frequently encountered domain, the reported “skills” involved in PO include the abilities to obtain tumor tissue, select the appropriate kit, and properly complete the ordering process:

*They need to know the difference between the tissue sample kits and the liquid biopsy kits. And they need to make sure that the kits aren’t expired.*

[Provider 1]

The majority of difficulties reported were in the latter category, with convoluted and unstandardized ordering processes sometimes creating difficulties with PO.

### Reinforcement: Motivation

As in the Goals domain, providers and patients noted an anticipated increase in the length or quality of life as a perceived positive consequence of PO, and providers feared missing this opportunity.

#### Treatment Outcomes

With regard to treatment outcomes, patients’ stated goals typically pertained to survival, with one patient stating: “Well basically it’s to stay alive” (Patient 51). Providers expressed similar goals as well as concerns about not meeting the goals. One provider stated:

*I’m thinking about targeted therapy so it’s a whole 15 to 17% of people you can deprive of potentially life prolonging medication. And the impact that you’re doing by not testing it, can you take it? Being an oncologist, treating oncology patients, can you take it with you every night and go to sleep? I cannot, so test for it.*

[Provider 3]

The negative consequences of treatment adverse effects were mentioned frequently in broad terms, but PO was seen as a way to potentially avoid these consequences.

#### Quality Outcomes

Respondents cited the benefits of selecting a more effective treatment as a positive outcome of PO and the detriment from the failure of PO.

### Social Influences: Motivation

Sources of motivation for patients and providers came from similar and distinct sources. This relationship was always dyadic in nature.

#### Patient-Provider

Patients frequently mentioned their providers as their principal source of advice on treatment-related issues. For example, one respondent stated, “Yes, yes. Total trust in my doctor” (patient 073). Comfort with and deference to providers emerged as common themes, whereas instances of conflict between patients and providers were mentioned only twice.

#### Patient-Other

Additional relevant social influences on patients come from their community, including family and friends.

#### Provider-Colleague

The most important social influence between providers appeared to be their peers’ consensus on the indications and use of PO:

*I think we’re constantly learning from each other. You know, we’re seeing our colleagues order testing in this setting and so we’re going to order.*

[Provider 5]

### Technical Challenges: Opportunity

How providers were able to operationalize PO treatments was the result of internal processes. These could facilitate or inhibit timely care delivery.

#### Ordering Process

This was the most frequently mentioned aspect of technical challenges. Providers noted frequent difficulties in completing the orders required to send testing and reported frequent breakdowns in communication with the pathology department related to initial processing. One respondent was quoted as saying:

*Trying to get the stuff ordered is more difficult than just ordering standard tests like immunohistochemistry or flow cytometry. We have to jump through a bunch of hoops for that and also the retrieval of the information. Once it’s done it requires a few phone calls, where’s my stuff?*

[Provider 13]

#### Specimen Acquisition or Processing

The expected procedural difficulties in obtaining a specimen were mentioned by several providers, and it was occasionally noted that tissue was available but at another institution. Knowing where kits were available and arranging for transport were also potential failure points, especially when dealing with peripheral blood NGS, obtained in the clinic.

#### Resulting Process

The principal issues with the resulting process were the long time it takes to generate a usable result and the inability to see the result once testing was completed.

### Conceptual Model

To illustrate the intersections of our results, we offer a conceptual model in Figure 1. Under the auspices of our overarching theme of “The Precision Oncology Feedback Loop,” the conceptual model illustrates the contribution of each of the areas of the BCW to the patient and provider interaction that ultimately influences PO implementation. Capability, opportunity, and motivation all influence and variously reinforce or negate each other, contributing to the implementation of PO.

## Discussion

### Principal Findings

Our study aimed to characterize the barriers and facilitators to PO. We found significant determinants of PO implementation at the patient, provider, and systems levels. In our analysis, the TDF provides a useful framework to conceptualize PO service implementation and, combined with the BCW, informs the design of interventions to improve PO implementation.

Our findings associated with system and operational issues are consistent with other studies. Prior research reported substantial structural or logistical issues in PO implementation, including coordinating or awaiting sample collection [25]. Our results add to previous work by presenting a more detailed analysis of specific factors associated with these problems, including interdepartmental communication–related barriers and the obstruction of interdepartmental communication via EHR ordering processes. Our data on the domains of Cognitive and Interpersonal Skills reiterated these challenges in communication. Furthermore, our finding about the role that staffing plays in PO care delivery, with a specific nod to the added benefit of patient navigators to help streamline test ordering, sample procurement, and results communication from pathology to providers, is novel.

From the patients’ perspective, our findings are associated with the social determinants of health around patient financial, logistics, and education specific to the VA population. The existing literature reports concerns around insurance coverage, high copays, and overall costs associated with treatment, which contrasts with our findings because VA participants do not bear those financial burdens [26]. This discrepancy is likely related to the VA single-payer model, which minimizes patient costs and third-party payer interactions, thereby eliminating a barrier to PO. Notably, existing literature is concordant with our findings that logistical challenges outside of cost (eg, transportation) represent barriers [26,27].

Patient education was perceived as a facilitator in our population, but only some patients noted receiving materials from their physicians, with many others turning to external resources. Several prior studies note that education can facilitate precision medicine when available and appropriately disseminated because it promotes patient engagement with care [28]. Yet, information sources sought by patients outside of their clinical team also suggest there is a risk for patients around misinformation in understanding precision medicine [26]. Our findings are also consistent with previous work identifying limited patient knowledge as a barrier to PO clinical trial accrual [28].

Regarding provider *Knowledge*, previous survey research reported that approximately 1 in 5 providers felt “low confidence” in their knowledge of clinical genomics and believed the tests would be used for less than 10% of patients [29]. Additional studies report that a lack of provider knowledge in genomic medicine may lead to delayed use [30]. Our findings reflect similar insecurities in providers regarding PO knowledge. Our data, however, build on previous literature by noting enthusiasm for systems-level educational interventions (ie, tumor boards noted as “immensely helpful”) and increasing comfort in interpreting NGS results (ie, providers feel “better in terms of outlining what’s pathogenic and what’s not”). Our data suggest a role for educational initiatives at the provider and patient levels to mitigate perceived knowledge gaps. Taken together, our findings along with the existing literature suggest that a deliberately implemented educational initiative may help facilitate PO uptake while also ensuring that patients do not fall victim to misinformation.

We noted that findings regarding the domains of Memory and Attention have substantial interplay with the findings in physician *Environment,* insofar as both relate to the structural/logistical burden that physicians face when implementing PO. Our findings were concordant with previous work suggesting barriers to precision medicine in time investment by physicians reviewing records, coordinating or awaiting sample collection, determining patient eligibility, and resolving insurance-related issues [26].

With regard to the domains of Goals and Reinforcement, previous studies similarly found that patients sought the benefits of PO clinical trials over previous less-targeted approaches to cancer care [31]. Our results support similar findings not only among patients considering clinical trials but also among all patients considering PO as standard care.

### Limitations

The limitations of our study include the following: (1) a small sample size comprising entirely of men, (2) VA-only study sites, (3) a risk of social desirability bias on the part of participants, and (4) bias in interpretation. Regarding sample size, our number of 33 (17 patients and 16 providers), although a relatively small cohort, was determined using theoretical saturation in which recruitment stopped when few additional new themes emerged. This approach increases efficiency while covering the majority of relevant themes. Of note, this is the largest sample of both patients and providers in any published work on the topic.

Regarding VA-only study sites, our study was conducted within the VA and represents viewpoints from a single, uniform health system. As such, external factors including insurance coverage, access to care, and access to trials are different from those in the private health care sector. Nonetheless, the VA setting does allow for geographic diversity and frequently samples patients with limited economic means, a population with well-reported underrepresentation in research [32,33].

A further consequence of VA-only sites is that our sample includes only male patients. The VA patient population is approximately 90% male, but the number of female patients is growing [34]. They may have unique PO treatment needs; thus, their perceptions may differ from those of their male counterparts. While we considered oversampling female patients, the cancers they are treated for are different from those of male patients and may change the PO oncology experience. Future research should focus specifically on female veterans to understand their needs with regard to PO.

We also chose to focus on the cancers that are most frequently treated in VA oncology clinics and to incorporate PO, that is, prostate, lung, and colorectal cancers, which yielded a predominantly male patient base. We made these decisions to focus on high-volume cancers, which can illuminate systemic challenges in a way that infrequently treated cancers cannot.

Finally, we recognize the constraints of social desirability bias along with biases in the interpretation of results. To mitigate social desirability bias, we maintained participant anonymity and confidentiality and used verbal and nonverbal cues to increase patient comfort [32]. To mitigate bias in interpretation, all interviews were coded independently by 3 reviewers, and disagreements on coding were resolved by discussion until consensus.

### Conclusion

In conclusion, our interviews with patients and providers added essential data to an understudied and quickly growing area of cancer care. Patients and providers reported barriers to Knowledge, Environment, Goals, and Attention. When mapped to the TDF or BCW, these identified barriers facilitate the design of a multilevel intervention for patients, providers, and systems to educate and improve the implementation of PO. Future research should engage with broader populations of specific cancers and also examine geographic variation more closely.

## Abbreviations

BCW: Behavior Change Wheel
IRB: institutional review board
NGS: next-generation sequencing
PO: precision oncology
TDF: Theoretical Domains Framework
VA: Veterans Affairs
VA-NYHHS: Veterans Affairs Medical Center – New York Harbor Healthcare System
VAMC: Veterans Affairs Medical Center
